# Bone Mineral Density in Congenital Generalized Lipodystrophy: The Role of Bone Marrow Tissue, Adipokines, and Insulin Resistance

**DOI:** 10.3390/ijerph18189724

**Published:** 2021-09-15

**Authors:** Erika Bastos Lima Freire, Catarina Brasil d’Alva, Mayara Ponte Madeira, Grayce Ellen da Cruz Paiva Lima, Ana Paula Dias Rangel Montenegro, Virginia Oliveira Fernandes, Renan Magalhães Montenegro Junior, Brazilian Group for the Study of Inherited and Acquired Lipodystrophies (BRAZLIPO)

**Affiliations:** Clinical Research Unit, Walter Cantídio University Hospital, Federal University of Ceará, Fortaleza 60416200, CE, Brazil; erikaendocrinologia@gmail.com (E.B.L.F.); cbdalva@terra.com.br (C.B.d.); mayara.madeira@hotmail.com (M.P.M.); grayceellen@yahoo.com.br (G.E.d.C.P.L.); apdrmontenegro@gmail.com (A.P.D.R.M.); virginiafernande@hotmail.com (V.O.F.)

**Keywords:** congenital generalized lipodystrophy, Berardinelli–Seip syndrome, bone, bone mineral density

## Abstract

Congenital Generalized Lipodystrophy (CGL) is a rare syndrome characterized by the almost total absence of subcutaneous adipose tissue due to the inability of storing lipid in adipocytes. Patients present generalized lack of subcutaneous fat and normal to low weight. They evolve with severe metabolic disorders, non-alcoholic fatty liver disease, early cardiac abnormalities, and infectious complications. Although low body weight is a known risk factor for osteoporosis, it has been reported that type 1 and 2 CGL have a tendency of high bone mineral density (BMD). In this review, we discuss the role of bone marrow tissue, adipokines, and insulin resistance in the setting of the normal to high BMD of CGL patients. Data bases from Pubmed and LILACS were searched, and 113 articles published until 10 April 2021 were obtained. Of these, 76 were excluded for not covering the review topic. A manual search for additional literature was performed using the bibliographies of the studies located. The elucidation of the mechanisms responsible for the increase in BMD in this unique model of insulin resistance may contribute to the understanding of the interrelationships between bone, muscle, and adipose tissue in a pathophysiological and therapeutic perspective.

## 1. Introduction

Congenital Generalized Lipodystrophy (CGL), also called Berardinelli–Seip syndrome, was first described in 1954 in two Brazilian children. Both patients had chronic diarrhea and hepatosplenomegaly and also presented muscle hypertrophy, acromegalic facies, changes in glycemic metabolism, and hyperlipidemia [1]. Five years later, Seip described three cases with the same clinical characteristics in Norway. In 1968, autosomal recessive transmission was suggested [2], and compound heterozygosity was found in many of those [3]. However, in rare cases, an autosomal dominant pattern was featured [4]. It is a rare disease and about 500 cases were reported since its first description [5]. The worldwide prevalence of 1:10,000,000 was estimated in 2004 [6]. 

CGL is characterized by the almost total absence of subcutaneous adipose tissue observed since birth or early childhood due to the inability of storing lipid in adipocytes. The extremely low adipose tissue is caused by mutations of genes responsible for adipocyte development [7]. Currently, the syndrome is described in four subtypes according to the corresponding mutations in four genes: *AGPAT2, BSCL2, CAV1,* and *CAVIN 1* [7]. The first two types, associated with mutations in *AGPAT2* and *BSCL2*, correspond to almost 95% of patients [5]

The clinical features include a generalized lack of subcutaneous fat, acromegalic facies, acanthosis nigricans, eruptive xanthomas, muscle hypertrophy (present in almost all individuals), phlebomegaly, umbilical protrusion, hepatomegaly, and polycystic ovary syndrome in girls after puberty [6,8,9]. Once adipose tissue is scarce, circulating adipokines (leptin and adiponectin) are low and patients evolve with an intense insulin resistance and poorly controlled diabetes mellitus as well as severe dyslipidemia [10].

CGL patients, especially *BSCL2* gene mutants, have a greatly reduced life expectancy. They evolve with severe metabolic disorders, non-alcoholic fatty liver disease, early cardiac abnormalities, and infectious complications, which can lead to death before the fourth decade of life [7,11].

The generalized lack of subcutaneous fat leads to normal or even low body mass index (BMI). Although low body weight is a known risk factor for osteoporosis, it has been reported that type 1 (CGL1) and 2 CGL (CGL2) have a tendency of high bone mineral density (BMD) [12,13,14]. Some mechanisms have been suggested, mostly based on the lack of the capacity to store fat in the peripheric adipose tissue and its consequences. Adiposity may influence bone turnover in different ways. The adipose tissue produces substances such as cytokines, growth factors, and adipokines that can affect skeletal homeostasis. Bone marrow fat also synthesizes factors able to influence bone cells [15,16]. Besides marrow adipose tissue and adipokines, such as leptin and adiponectin, other probable mechanisms involved in CGL high BMD are muscle hypertrophy, hyperinsulinemia, low sexual hormone binding globulin (SHBG), and osteoarthritic degeneration [17,18]. In this review, we discuss the role of bone marrow tissue, adipokines, and insulin in the setting of the normal to high BMD of CGL patients.

## 2. Materials and Methods

Data bases from Pubmed and LILACS were searched using the following keywords “bone density” or “bone” or “bone mineral density” or “bone mineral content “and “congenital generalized lipodystrophy” or “Berardinelli–Seip syndrome” or “Congenital generalized lipodystrophies” with interposition of the Boolean operator “AND”. In total, 113 articles published until 10 April 2021 were obtained. Of these, 76 were excluded because they did not contemplate the theme of the review. A manual search for additional studies was performed using the bibliographies of the reports and reviews located. Only articles in English were considered.

## 3. Discussion

### 3.1. Marrow Adipose Tissue (MAT)

Normal bone marrow is constituted by a variable proportion of hematopoietic cells and adipocytes. A physiological bone marrow conversion, in which it gradually shifts from red to fat cells, occurs from birth to adulthood, in a centripetal pattern [19,20]. Adipocytes are one of the most abundant cell types in bone marrow at age 25, accounting for approximately 70% of human bone marrow [21]. Their distribution varies with body site, being more prevalent on the appendicular skeleton, where MAT accumulation starts on childhood from distal to proximal regions until age 25. On the other hand, the transformation continues in the axial skeleton throughout life [13].

The adipocytes are now considered an essential part of the bone marrow micro-environment. MAT increase is observed not only in aging, but also in many bone diseases, such as osteoporosis [15,22,23,24]. Both adipocytes and osteoblasts originate from a common pluripotent mesenchymal stem cell. They compete with each other, promoting either adipogenesis or osteogenesis [25,26,27]. Thus, MAT can be considered a negative regulator of bone formation and, in clinical studies, it has been inversely associated with BMD [21]. It was demonstrated that not only mesenchymal progenitors, but also mature cells can differentiate into either osteocytes or adipocytes, showing an intrinsic relationship between bone and MAT [28].

An early report of skeletal abnormalities of three CGL patients showed a lack of fat in bone marrow and evidenced its occupation with vascularized tissue [29]. The MRI findings posteriorly described diffuse serous transformation of bone marrow fat, which is consistent with adipocyte atrophy [30]. The CGL gene mutations have different effects in endocrine and mechanical white adipose tissue and MAT, which is absent or low in CGL 1 and 2 and maintained in type 3 (CGL 3) and 4 (CGL 4) [31]. Since the latter two have preserved MAT and a tendency of decreased BMD, the absence of bone marrow adipocytes in CGL 1 and CGL 2 could favor osteoblast proliferation, contributing to osteosclerosis and a tendency of high BMD in these patients [30]. Bone formation also increased in mice with severe lipodystrophy induced by a knock-out of peroxisome proliferator-activated receptor gamma (PPARγ) in white fat and the inverse correlation between bone density and MAT was also observed in leptin-deficient mice [28,32]. They had lower BMD in cortical bone and a marked increase in MAT compared to the controls. On the other hand, in trabecular zones (with higher BMD), only a few adipocytes were observed in bone marrow [33]. This inverted correlation could strengthen the hypothesis that MAT might play an important role in the etiology and pathogenesis of the tendency of high bone mass in CGL 1 and CGL 2 patients.

### 3.2. Leptin

Since the discovery of leptin as a product of adipose tissue in 1994, it has been considered a contributor to energy metabolism, and an interrelationship between bone and fat has been demonstrated [34]. The adipose tissue synthesizes several cytokines involved in bone metabolism, with leptin being an important hormone in this process.

The first studies of the effect of leptin in bone were performed in an animal model. Ducy et al. [35] showed that severely obese *ob/ob* mice, carriers of an inactivating mutation of the leptin gene, had increased bone mass in trabecular sites (with normal cortical bone), despite hypogonadism and hypercortisolism. An intracerebroventricular leptin infusion led to rapid bone loss, which suggested leptin as an important negative regulator of bone formation through a central pathway [35]. Posterior studies were conflicting and, sometimes, had opposite results. Contrasting with prior studies, intraperitonially administered leptin was found to be a potent stimulator of bone growth in the *ob/ob* mice with short stature [36]. The investigation of the effect of leptin on bone architecture in *ob/ob* mice found that it does not act uniformly throughout the skeleton and differs greatly between the axial and appendicular skeleton. In this study, leptin-deficient mice also had increased BMD in trabecular zones, but decreased cortical thickness in both the spine and femur was observed [33]. These outcomes in animal studies have been justified by different actions of leptin on the skeleton, depending on the bone site, and suggest that, possibly, cortical and trabecular areas respond differently to leptin. In the first animal studies, the infusion of leptin into the third ventricle showed an indirect and resorptive effect in bone [35,37]. This adipokine binds to the hypothalamic receptors, increasing sympathetic activity, which stimulates beta-2 adrenergic receptors on osteoblasts. This causes the inhibition of osteoblastic proliferation (sympathetic activation stimulates expression of *Esp*, a gene that inhibits the activity of osteocalcin) and increases RANKL, promoting osteoclastic trabecular bone resorption [33,34,38]. This indirect and negative effect, first observed by Ducy et al. [35] in *ob/ob* mice, has not been demonstrated in humans, showing that there are differences in leptin actions among species [37,39]. More recently, Turner et al. [40] suggested that the effects of leptin could depend not only on bone compartment (trabecular vs. cortical), once they observed a site-independent increase in osteoblastic activity when leptin was subcutaneously administered, contrasting with previous studies. They suggested that mice age and gender could be factors to justify these findings. The authors proposed that once subcutaneous leptin crosses the blood brain barrier, its peripheral effects should not distinguish from the central actions of leptin on bone [40]. On the other hand, it has been reported that, in vitro, the human recombinant leptin has an anabolic effect. It directly stimulates the proliferation and differentiation of marrow mesenchymal cells to the osteoblastic pathway with the inhibition of late adipocytic differentiation [41].

Despite the evidence from experimental studies, in which many showed a great impact of leptin on bone metabolism, its possible role in skeletal turnover was not confirmed in the few studies conducted in leptin-deficient humans [42]. Similar to animal models, in which mice with a lack of leptin had increased bone mass, CGL 1 and CGL2 patients have a tendency of a high BMD with a higher mineral content in trabecular zones [17,18]. Instead, the replacement of leptin in humans did not have impact on bone mass, as it did in some animal research, in which intracerebroventricular infusion of leptin had a negative effect on bone mass [18,35,43,44,45]. Subcutaneous recombinant leptin was unable to change the BMD of 31 CGL 1 and CGL 2 patients [18]. Conversely, hypoleptinemic women with hypothalamic amenorrhea were found to have low BMD, demonstrating that the lack of leptin is not the only reason in which CGL may have high BMD, but an improvement in BMD after long-term metreleptin (recombinant leptin) administration shows that it may play a role [46].

Since hypoleptinemia can also regulate the production of neuroendocrine hormones (many anabolic), it is difficult to isolate and assess the effects of leptin only on the skeleton, especially in human studies [37,47,48]. There are many reports on the interrelationship of leptin and sex hormones. Scheller et al. [13] compiled 24 reports in humans and observed that leptin often correlates positively with BMD in females and negatively in males. The mechanism was not demonstrated. In addition, the presence of different forms of CGL in human studies makes the analysis of leptin’s real role in CGL bone homeostasis more difficult.

To conclude, according to Reid et al. [34], the prevalent leptin effect on bone (anabolic x catabolic) depends on several aspects such as species, gender, individual factors such as BMI, medical or even hormonal disorders; as well as leptin levels and its resistance. In light of currently available information, we cannot rule out that this adipokine can somehow regulate bone metabolism.

### 3.3. Adiponectin

As mentioned, CGL patients have low leptin and adiponectin due to the lack of fat [10]. To understand whether adipose tissue, by itself, regulates bone homeostasis, Zou et al. [49] generated mice without visceral, subcutaneous, and visible brown fat, but with preserved bone marrow adipocytes. Despite preserved bone marrow, their bone evaluation revealed osteosclerosis, which is consistent with increased skeletal mass due to heightened osteogenesis. The subcutaneous transplantation of mouse embryonic fibroblasts, which differentiate into white tissue fat, completely normalized their bone. Once bone marrow adipocytes were preserved, it was proposed that the lack of white and brown adipose tissue caused osteosclerosis. While this animal model mimics the phenotype of CGL patients, there must be caution to extrapolate the results of the study to humans, since CGL has a different pathogenesis. On the other hand, the bone effect in this report suggests that substances produced by the adipocytes, such as leptin and adiponectin, have an effect on the skeleton [49]. This last adipokine is abundantly present in plasma and highly expressed in fat (visceral, subcutaneous, and bone marrow adipocytes). Adiponectin and its receptor have also been identified in osteoblasts and osteoclasts, although in much lower expression than in fat [50,51,52,53].

The effects of adiponectin on bone metabolism are complex. The results of in vivo and in vitro studies are conflicting. Most in vivo research suggests a negative bone effect while most in vitro studies propose the contrary, osteoblast proliferation [50,54]. Yokota et al. [16] stated that adiponectin can inhibit the differentiation of cloned stromal preadipocytes in bone marrow, favoring osteogenesis. On the other hand, another experimental study showed that it may affect osteoclast activity, induce RANKL, and inhibit osteoprotegerin expression in human osteoblasts [55].

In most in vivo studies, a negative effect consistent with osteoblast suppression was observed, with contrasting results from the research in vitro [56]. Kajimura et al. [57] studied the role of adiponectin on bone homeostasis of mice and proposed that it regulates bone mass through two opposite mechanisms, one local and the other central. It was found that it acts directly and negatively on bone by promoting osteoblast apoptosis and indirectly by decreasing sympathetic nervous system activity, which counteracts part of leptin’s negative effect [57]. Evidence shows that the effect of adiponectin is complex and can vary not only with the two mechanisms just described. Shinoda et al. [53] studied bone effects of adiponectin separately via autocrine/paracrine and endocrine pathways and also found opposite actions. It was demonstrated a direct and negative action on the osteogenesis by circulating (systemic) adiponectin, which also promoted a positive regulation via amplification of insulin signaling and, consequently, an increase in osteocalcin production [58]. Many studies have reported that adiponectin enhances insulin activity, and hyperinsulinemia was already described as a possible mechanism of high BMD in CGL [17,53]. On the other hand, the autocrine/paracrine pathway by adiponectin within the bone marrow had positive effects on the skeleton [52,53].

Based on current knowledge, it has been proposed that the predominant effect, in vivo, depends on if it acts directly or indirectly on bone or through autocrine/paracrine or endocrine pathways, and most studies are consistent with osteoblast suppression [56]. In humans, as in most in vivo studies, an inverse association of adiponectin with BMD has been shown [51,59,60]. Xinyan et al. [61] reported recently a sex-dependent effect of adiponectin in humans, similar to leptin. It was correlated with low BMD in females, but not in males. It is possible that the low adiponectin levels could influence bone homeostasis of CGL patients and help to explain their tendency of high BMD, although more studies are needed to confirm this hypothesis.

### 3.4. Insulin Resistance

CGL individuals have intense insulin resistance and consequently hyperinsulinemia. Many evolve with diabetes mellitus (DM). Type 2 DM also presents normal to high BMD [62]. This finding can result from the positive effects of insulin and overweight on bone. It has been proposed that insulin is an anabolic agent for bone [63,64]. It may affect bone homeostasis directly by modulating actions of bone cells and indirectly by mainly increasing anabolic hormones. Hyperinsulinemia causes hyperandrogenism through elevation of sex hormone free fractions due to decreased SHBG (sexual hormone binding globulin), increased hepatic insulin-like growth factor-1 (IGF-1) production, and/or increased IGF-1 bioavailability through a reduction in IGFBP-1 generation [17,63]. Insulin acts directly on bone through the insulin receptor, which is expressed in both osteoblasts and osteoclasts [64]. In vitro studies have shown that insulin decreases osteoclast activity by reducing the RANKL signaling pathway, but stimulates osteoblasts, favoring osteogenesis [65]. Fulzele et al. [58] demonstrated that an insulin receptor is required for osteoblast proliferation, survival, and differentiation, and suppresses the inhibitor of Runx2, a transcription factor determinant of osteoblast differentiation. The author also showed that insulin induces osteocalcin production, the most abundant osteoblast-specific protein and a determining factor of bone formation [58,66]. Besides, there is also a positive feed-back since osteocalcin increases insulin secretion and sensitivity.

In animal studies, it was shown that insulin resistance, in which insulin levels are supraphysiological, leads to decreased bone turnover and therefore, a tendency of a high BMD [65,67,68]. Indeed, it has been reported that biochemical markers of bone formation and resorption are decreased in type 2 DM and insulin resistance, independent of adiposity [58,64,65]. However, this state of low-turnover leading to a high BMD also contributes to bone fragility [64]. Lima et al. [17] proposed that hyperinsulinemia, due to CGL severe insulin resistance, may exacerbate anabolic insulin mechanisms on bone, possibly causing the tendency of high BMD. However, CGL3 and 4 also present hyperinsulinemia and still have low BMD, which suggests that insulin resistance alone is not the main mechanism of high BMD in CGL1 and CGL2 [13]. Its effects on bone are not yet fully elucidated. We can speculate that the high BMD is merely the consequence of a low turnover state caused by the insulin-resistance in bone, or, on the other hand, that bone is not an insulin-resistant tissue and, therefore, is sensible to insulin anabolic effects. Since most evidence comes from experimental studies, more human research is needed to help understand this mechanism.

### 3.5. Cross Talk between Mat, Adipokines, Insulin, and Bone

CGL is a condition marked by its severe insulin resistance. Many clinical and cellular aspects of this rare disease are yet to be studied and understood. The elucidation of the mechanisms responsible for the increase in BMD in this unique model of insulin resistance may contribute to the understanding of the interrelationships between bone, muscle, and adipose tissue from a pathophysiological and therapeutic perspective. However, precise mechanisms are not known.

The inverse correlation between BMD and MAT seems to play a role. Leptin, adiponectin, and hyperinsulinemia are also probably involved. Adipokines have a complex interrelationship with the other hormones; their effects depend on many other aspects including the site of action and some mechanisms involved, such as the ones occurring in the bone marrow. As cited above, hyperinsulinemia can increase IGF-1 production. It is produced mainly in the liver by the growth hormone (GH) command, and can also be produced by osteoblasts, enhancing its activity and favoring bone formation [28].

MAT was recently recognized as a part of the endocrine system, since it was found that it secretes adipokines directly in the bone marrow micro-environment, influencing osteoblast function [28,69,70]. Adiponectin in bone marrow cultures favors osteogenesis by inhibition of the differentiation of cloned stromal preadipocytes [16]. It was also demonstrated, in vitro, that bone marrow adipocytes secrete leptin and that its receptor can be found in adult primary osteoblasts and chondrocytes, probably contributing to bone formation [40,71]. The effect of leptin in bone marrow preosteoblasts is anabolic, therefore different from the central action reported for this hormone. Besides leptin and adiponectin, other factors can influence osteoblast differentiation and function in the bone marrow environment. Increased PPARγ expression, caused by oxidative stress and excess glucocorticoid use, for example, and oestrogen insufficiency promote adipogenesis, negatively affecting bone mass [28,72]. On the other hand, 1,25-dihydroxyvitamin D3 and IGF-1 may lead to a decrease in PPARγ2 expression, contributing to bone formation [28].

### 3.6. Bone Mineral Density in CGL

Since CGL is a rare disease, there are a few human studies about its skeletal features. Most patients reported are CGL1 and CGL2, since they account for 95% of cases [5]. Unfortunately, the data on CGL 3 and 4 are very scarce; only about 500 CGL patients have been reported so far [5]. Therefore, most studies describe a high BMD among these patients. However, some case reports were published before genotyping was available and there are only a few series of cases in the literature so far [14]. Christensen et al. [18] studied the bone of 31 CGL 1 and CGL 2 patients (10 BSCL2, 21 AGPAT2) that received subcutaneous recombinant leptin. At baseline, total body less head BMD was increased. Patients had normal 25-hydroxyvitamin D, osteocalcin, parathormone, alkaline phosphatase, serum phosphate and calcium, estradiol, and testosterone levels. Insulin was increased. The high BMD was assigned to the high lean mass and tall stature of the patients [18].

To our knowledge, there are no studies evaluating the pitfalls on DEXA BMD in patients with an absence of marrow fat. However, studies on CGL bone radiological findings describe bone sclerosis and cortical thickening, especially in the axial skeleton and could suggest that CGL 1 and 2 BMD have a tendency to be higher.

Scheller et al. [13] performed an extensive review of longitudinal studies and standard case reports in which some kind of skeletal analysis of CGL patients was performed. The author divided the individuals into two subgroups. The first one was composed of 75 CGL 1 and 2 as well as patients with unknown CGL-causing genetic mutations, and the other subgroup was composed of 18 CGL 3 and 4 patients. Until 10 years of age, the first subgroup presented accelerated bone growth, advanced skeletal age, cortical thickening, and osteosclerosis (characterized by an increase in skeletal mass that can be focal or diffuse). After the first decade of life, some patients developed cystic lesions in the long bones and maintained increased BMD as well as osteosclerosis. The subgroup with CGL 3 and CGL 4 had a tendency of decreased BMD after the first decade of life and did not have evidence of sclerosis or cysts [13]. Table S1 in Supplementary Materials summarizes the status of Adipokines, Insulin Resistance, MAT and BMD among Congenital Generalized Lipodystrophies subtypes.

Lima et al. [12] evaluated 21 CGL subjects (15 *BSCL2,* 3 *AGPAT2*, 3 not genotyped) and reported that 57% of those had high BMD, despite physical inactivity, delayed menarche, low vitamin D levels, BMI, and daily calcium intake in the majority of patients. Serum calcium and phosphate were both in the normal range. In this study, for the first time, it was reported that BMD was higher in trabecular than in cortical bone compartments. Eleven of those patients were also evaluated regarding sclerostin, a negative regulative of bone formation, and Trabecular Bone Score (TBS), a densitometric tool used to assess trabecular microarchitecture and fracture risk. Eight patients had normal TBS, which is consistent with normal microarchitecture. Three females had partially degraded microarchitecture. Despite these good results in the majority of patients, ten subjects had high sclerostin, showing that this hormone does not play a major role in CGL‘s BMD [73].

## 4. Conclusions

In conclusion, CGL1 and CGL2 patients lack MAT and have very low adipokine levels and hyperinsulinemia due to severe insulin resistance. Possibly, all these factors have a complex interrelationship and may contribute to the increased osteogenesis in CGL1 and CGL2 patients, although, the results of adipokine studies are controversial, and patients with CGL3 and CGL 4, which also have insulin resistance, have low BMD. More studies are needed to further evaluate the precise mechanisms of bone outcomes in this rare disease.

## Supplementary Materials

The following are available online at https://www.mdpi.com/article/10.3390/ijerph18189724/s1, Table S1: Adipokines, Insulin Resistance, MAT and BMD in Congenital Generalized Lipodystrophies subtypes.
