# Supplementary material for: Bone Mineral Density in Congenital Generalized Lipodystrophy: The Role of Bone Marrow Tissue, Adipokines, and Insulin Resistance
Source: Int J Environ Res Public Health. 2021 Sep 15;18(18):9724. doi: 10.3390/ijerph18189724 (PMC8465110; doi:10.3390/ijerph18189724)
Supplement: Supplementary file 1 [file ijerph-18-09724-s001.zip › ijerph-1276244-supplementary.pdf]

Table S1 - Adipokines, Insulin Resistance, MAT and BMD in Congenital Generalized Lipodystrophies subtypes

Subtypes (mutation)	Leptin	Adiponectin	Insulin resistance	MAT	BMD
**CGL1** *AGPAT2*	Low	Low	Severe	Absent or low	High
**CGL2** *BSCL2*	Low	Low	Severe	Absent or low	High
**CGL3** *CAV1*	Low	Low	Severe	Preserved	Decreased
**CGL4** *CAVIN 1*	Low	Low	Severe	Preserved	Decreased

BMD = Bone Mineral Density; MAT = Marrow Adipose Tissue; CGL = Congenital Generalized Lipodystrophy
